# A co-ordinated interaction between CTCF and ER in breast cancer cells

**DOI:** 10.1186/1471-2164-12-593

**Published:** 2011-12-05

**Authors:** Caryn S Ross-Innes, Gordon D Brown, Jason S Carroll

**Affiliations:** 1 Cancer Research UK, Cambridge Research Institute, Li Ka Shing Centre, Robinson Way, Cambridge, CB2 0RE, UK; 2 Department of Oncology, University of Cambridge, Cambridge CB2 0RE, UK

## Abstract

**Background:**

CCCTC-binding factor (CTCF) is a conserved zinc finger transcription factor that is involved in both intra- and interchromasomal looping. Recent research has shown a role for CTCF in estrogen receptor (ER) biology, at some individual loci, but a multi-context global analysis of CTCF binding and transcription activity is lacking.

**Results:**

We now map CTCF binding genome wide in breast cancer cells and find that CTCF binding is unchanged in response to estrogen or tamoxifen treatment. We find a small but reproducible set of CTCF binding events that overlap with both the nuclear receptor, estrogen receptor, and the forkhead protein FOXA1. These overlapping binding events are likely functional as they are biased towards estrogen-regulated genes, compared to regions lacking either CTCF or ER binding. In addition we identify cell-line specific CTCF binding events. These binding events are more likely to be associated with cell-line specific ER binding events and are also more likely to be adjacent to genes that are expressed in that particular cell line.

**Conclusion:**

The evolving role for CTCF in ER biology is complex, but is likely to be multifunctional and possibly influenced by the specific genomic locus. Our data suggest a positive, pro-transcriptional role for CTCF in ER-mediated gene expression in breast cancer cells. CTCF not only provides boundaries for accessible and 'protected' transcriptional blocks, but may also influence the actual binding of ER to the chromatin, thereby modulating the estrogen-mediated gene expression changes observed in breast cancer cells.

## Background

Estrogen receptor alpha (ER), the driving transcription factor of the majority of breast cancer tumors, is a nuclear receptor that binds to the chromatin in order to regulate transcription of its target genes, ultimately to promote cell proliferation. ER most frequently binds to enhancer regions and rarely to promoter regions [1,2], and ER binding to the chromatin has been shown to require the pioneer factor, FOXA1 [2-5]. In addition to the pioneering function of FOXA1 for interaction with condensed chromatin, ER also requires a host of cofactors in order to regulate gene transcription of its target genes. Transcription involves chromatin loops that form between ER bound to enhancer regions and promoter regions of target genes [6,7].

There has been recent interest in understanding the possible role of the insulator protein, CCCTC-binding factor (CTCF) in ER biology. CTCF is a highly conserved and abundant zinc-finger protein that is ubiquitously expressed in the majority of tissue types. It is a large protein including 11 zinc fingers which it uses to bind to the DNA. CTCF was originally identified as a transcription factor that binds to the mammalian and avian MYC promoter [8-10]. More recently many different roles have been attributed to CTCF: it has now been identified as a transcriptional activator [11], a transcriptional repressor [8], a transcription factor involved in hormone-responsive gene silencing [12,13], an insulator protein [14], a protein involved in imprinting [15] and X-chromosome inactivation [16] as well as a participant in long-range chromatin interactions, both within and between chromosomes [17].

As the binding profiles of CTCF and ER have now been published [1,2,5,18-22], several studies have endeavoured to understand potential interactions between CTCF and ER. Initially, computational methods were employed to describe the global pattern of ER and CTCF binding events [23]. Chan and Song proposed that CTCF binding partitions the genome into ER-regulatory blocks that contain ER binding events and estrogen-regulated genes. This initial observation was validated on the TFF1 locus, which showed that CTCF can demarcate regions of the genome that are responsive to estrogen treatment [24]. Two CTCF binding events flanking the TFF1 locus were shown to act as boundary elements by preventing the spread of heterochromatin and allowing the genes within this region to be estrogen regulated.

It is currently unknown what the global role of CTCF is in estrogen and tamoxifen-mediated gene transcription in breast cancer cells. We show on a genome-wide scale that CTCF binding is static in breast cancer cells in response to estrogen or tamoxifen treatment. We show that CTCF co-localises with key transcription factors in breast cancer cell lines and that these co-bound regions are likely to be functional. We identify cell-line specific CTCF binding events in different breast cell lines; these cell-line unique CTCF binding events are associated with genes that are highly expressed in that cell line.

## Results and discussion

### CTCF binding is static in response to estrogen or tamoxifen treatment

**Figure 1:**
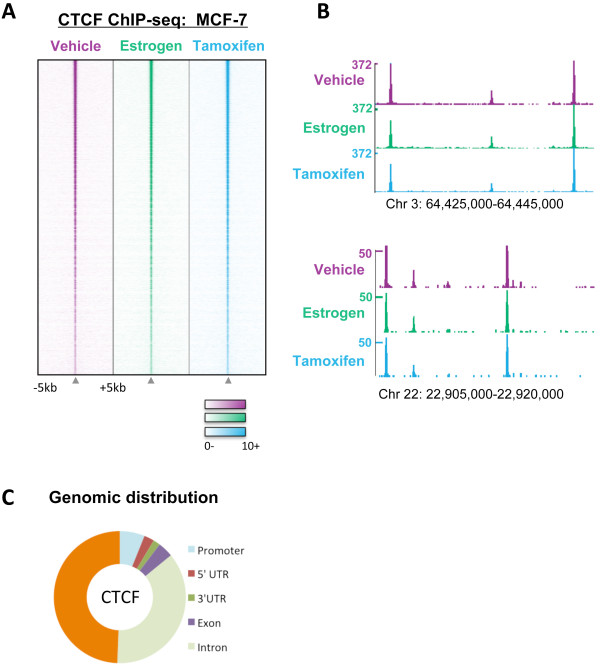
**CTCF binding does not change in response to estrogen or tamoxifen treatment**. CTCF binding was mapped by ChIP-seq in MCF-7 cells treated with vehicle, 100 nM estrogen or 1 μM tamoxifen for 45 minutes and 3 hours. **A**. Heat map representing CTCF binding intensity in MCF-7 cells treated with vehicle, estrogen or tamoxifen for 45 minutes. The window represents -/+ 5 kb regions from the centre of the CTCF binding events. **B**. Examples of genomic loci showing CTCF binding in vehicle-, estrogen- and tamoxifen-treated MCF-7 cells. **C**. Global genomic distribution of CTCF binding events in MCF-7 cells.

CTCF is a ubiquitously expressed protein that has been well documented to act as an insulator protein and prevent looping between enhancers and promoters [14,25]. Previous reports have demonstrated that looping between ER and promoters of estrogen-regulated genes is required for estrogen-mediated transcription of target genes [6,7,26]. We therefore hypothesised that CTCF binding may play a role in regulating ER gene transcription by preventing transcription of estrogen target genes in the presence of tamoxifen. To test this hypothesis, MCF-7 cells were hormone deprived for three days and then treated with vehicle, 100 nM estrogen or 1 μM tamoxifen for 45 minutes and three hours. Genome-wide CTCF chromatin immunoprecipitation followed by sequencing (ChIP-seq) was performed; in all treatments and time points at least seven million aligned reads were obtained (Additional File 1). Peaks were called using MACS [27] and at least 56, 342 CTCF binding events could be identified across the genome in all the samples (Additional File 1). The overlap between the samples was at least 91% and no reproducible differences were observed in the different treatment conditions (Figure 1A and 1B) at any of the time points. Genomic distribution analysis showed that CTCF binding in MCF-7 cells occurred mostly at intergenic and intronic regions, with only 5.7% CTCF binding events occurring within 1 kb of promoters (Figure 1C).

CTCF binding has previously been shown to separate the genome into different blocks, some which contain ER binding regions and ER-regulated genes, and some which do not [23]. Similar patterns were observed in this study (Additional File 2) suggesting that CTCF may be required at these regions to demarcate the estrogen-responsive genes within the chromatin.

### CTCF binding can co-localise with ER and FOXA1 binding

As the CTCF motif has previously been shown to be enriched in ER binding regions [28], we asked whether CTCF binding in MCF-7 cells overlaps with ER binding. In addition, we assessed whether CTCF binding overlaps with the pioneer factor FOXA1, which has been shown to be required for ER binding to the chromatin and proliferation of ER-positive cells [2-5]. As we have shown that CTCF binding does not change with estrogen or tamoxifen treatment, CTCF binding in hormone-deprived, vehicle-treated MCF-7 cells was used for the analysis. Considering peaks that were called in both replicates, 55, 176 CTCF binding events could be identified across the genome. ER binding was also mapped in proliferating MCF-7 cells, in duplicate, resulting in 57, 662 ER binding events (Additional File 1). For the FOXA1 binding data, a previously published dataset was used that identified 79, 624 FOXA1 binding events in vehicle-treated MCF-7 cells [5]. The FOXA1 ChIP-seq was conducted and analysed in exactly the same way as the CTCF and ER ChIP-seq data.

**Figure 2:**
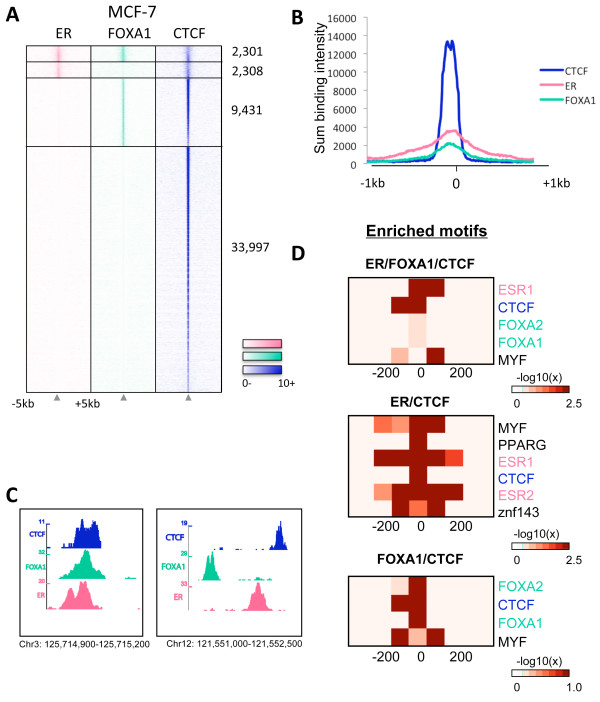
**CTCF binding can co-localise with ER and/or FOXA1 in MCF-7 cells**. CTCF, ER and FOXA1 binding profiles in MCF-7 cells were analysed. **A**. Heat map showing clustered binding signal for ER, FOXA1 and CTCF binding in MCF-7 cells. The heat map shows regions co-bound by ER/FOXA1/CTCF, or ER/CTCF or FOXA1/CTCF. The window represents -/+ 5 kb regions from the centre of the CTCF binding events. **B**. The sum of the normalised binding intensity profile of CTCF, ER and FOXA1 in regions co-bound by all three factors is shown. **C**. Two examples of genomic loci bound by CTCF, ER and FOXA1 in MCF-7 cells. **D**. Heat maps showing enriched motifs (the p values are shown) in regions bound by CTCF together with ER and/or FOXA1.

Using the definition that a binding region must occur in both replicates, overlapping by at least one base pair, almost a third (29%, 14, 040/48, 037) of CTCF binding events co-localised with ER and/or FOXA1 (Figure 2A), with the majority (9, 431/14, 040) of these co-localised binding regions consisting of FOXA1 and CTCF binding, but not ER binding. The probability of this overlap occurring by chance is p ≈ 0 using the GSC method [29,30]. Interestingly, when considering the 2, 301 genomic loci bound by CTCF, ER and FOXA1, the binding of ER and FOXA1 did not necessarily occur directly over the CTCF binding summit, but did occur within 1 kb of the CTCF binding summit (Figure 2B). Due to the non-centred nature of the ER and FOXA1 binding in the heat map and intensity plot, the ER and FOXA1 binding appear weaker in the regions bound by CTCF/ER/FOXA1. However, the normalised sum of the intensities of CTCF, ER and FOXA1 binding, in regions co-bound by all three factors, was 190, 782, 252, 928 and 137, 374, respectively, showing that ER binding intensity at these regions is not weaker than CTCF binding.

To determine whether ER and FOXA1 are binding directly to the DNA at regions co-bound by CTCF, ER and/or FOXA1, motif analysis was performed. The data shows that regions bound by CTCF/ER/FOXA1 are enriched for estrogen response elements (ERE), CTCF and forkhead motifs, suggesting that all three proteins bind to the DNA at these regions (Figure 2D). Similarly, regions bound by ER/CTCF were significantly enriched for ERE and CTCF motifs, and regions bound by FOXA1/CTCF were enriched for CTCF and forkhead motifs, although the enrichment of these motifs was not significant using this stringent motif analysis. In regions only bound by CTCF/FOXA1 but not ER, no enrichment for ERE motifs was detected and in regions bound by only CTCF/ER, no forkhead motifs were enriched. Interestingly, the ERE motifs were enriched in a large window surrounding the summit of the peaks, especially in the ER/CTCF co-bound regions. This is in line with the ER binding data that is not centred over the CTCF binding summit. The only other motifs that were statistically enriched in these categories were MYF, znf143 and PPARG, although it is currently unclear what the significance of these motifs is.

To determine whether these observations were unique to MCF-7 cells, CTCF and ER binding were mapped in another ER-positive cell line, ZR75-1. ChIP-seq was performed in duplicate for both factors and at least 19 million mapped reads were obtained per library (Additional File 1). Considering peaks that were called in both replicates, 41, 683 ER binding events and 48, 898 CTCF binding events could be identified in the ZR75-1 cells. Previously published data reporting 74, 670 FOXA1 binding events in ZR75-1 cells was also used [5]. Overlapping of the datasets revealed 4, 023 regions bound by ER/FOXA1/CTCF or ER/CTCF or FOXA1/CTCF (Additional File 3). The majority (60%) of the 4, 023 regions co-bound in the ZR75-1 cell line were also co-bound in the MCF-7 cell line, perhaps indicating a conserved function.

Genomic location analysis in the MCF-7 cell line revealed the striking result that 21.7% of the ER/CTCF regions were located within 1 kb upstream of transcriptional start sites. This differs from a normal ER binding profile as ER binds predominantly in enhancer regions and rarely at promoter regions (< 5% ER binding events are within 1 kb promoter regions) [1,2]. Furthermore, 11.4% of the ER/FOXA1/CTCF bound regions were located within one kb of promoter regions. However, the CTCF unique and FOXA1/CTCF regions displayed a normal CTCF genomic distribution with about 5% of binding events occurring within 1 kb promoter regions. The genomic distribution analysis of the ZR75-1 data differed in that all the different categories displayed a normal CTCF distribution, with between 3 and 5.6% of the CTCF binding events occurring at promoter regions.

### CTCF and ER co-bound regions are enriched near estrogen-regulated genes, compared with regions lacking either one

To determine whether the regions bound by CTCF/ER and/or FOXA1 are likely to be functional and involved in regulating gene transcription, the binding data in MCF-7 was overlapped with a previously published gene expression dataset that identified genes that are up or down regulated after estrogen stimulation [1]. In order to assess the direct transcriptional effects of ER, early time points were used for the analysis, namely three and six hours after estrogen treatment. Any genes that significantly changed (p < 0.01) at either time point were included in the analysis. This resulted in the identification of 1, 608 estrogen-upregulated genes, and 1, 350 estrogen-downregulated genes. As ER most often binds to enhancer regions, a 20 kb window on either side of the transcriptional start site of the genes was assessed for CTCF, ER and FOXA1 binding events (a 20 kb window has been previously identified from cell line experiments as an appropriate window between ER binding events and regulated genes [31]).

**Figure 3:**
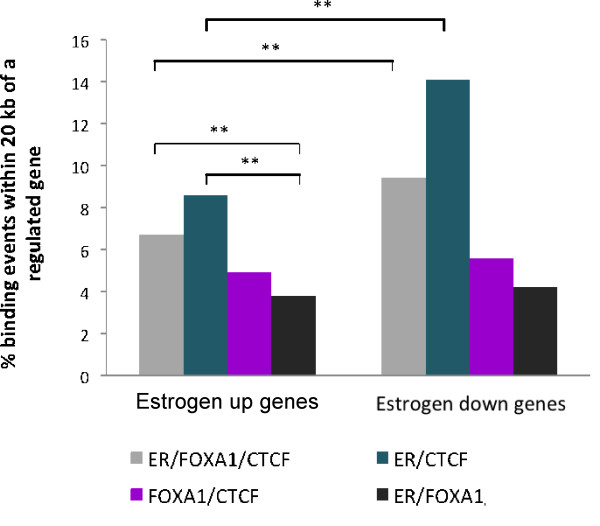
**CTCF/ER bound regions are more likely to be near estrogen-regulated genes**. Genomic regions bound by ER/FOXA1/CTCF, ER/CTCF, FOXA1/CTCF or regions bound by ER and FOXA1, but not CTCF, were analysed to determine whether they were enriched near estrogen-regulated genes. Genes that are up- or down-regulated by estrogen within six hours of estrogen treatment were included. The percentage of binding events +/- 20 kb from an estrogen up- or down-regulated gene was assessed. Graph showing the percentage of binding events in the different classes that are -/+ 20 kb from estrogen up- or down-regulated genes. Included as a control are binding events that overlap between ER and FOXA1 binding but do not overlap with CTCF binding. ** denotes p < 2.5 × 10^-5^

Results showed that regions bound by ER and CTCF were, on the whole, more likely to be near estrogen-regulated genes, and specifically estrogen-downregulated genes, when compared with regions bound by either one (Figure 3). ER/FOXA1/CTCF binding events were also significantly biased towards estrogen-regulated genes, compared to ER/FOXA1 binding events that do not have an overlapping CTCF binding event. These results suggest that CTCF can mark euchromatic regions, which may allow ER to bind and then activate or repress expression of its target genes. Surprisingly, regions bound by ER and FOXA1, but not CTCF, were least likely to be near estrogen-regulated genes.

### CTCF can bind uniquely in breast cell lines

**Figure 4:**
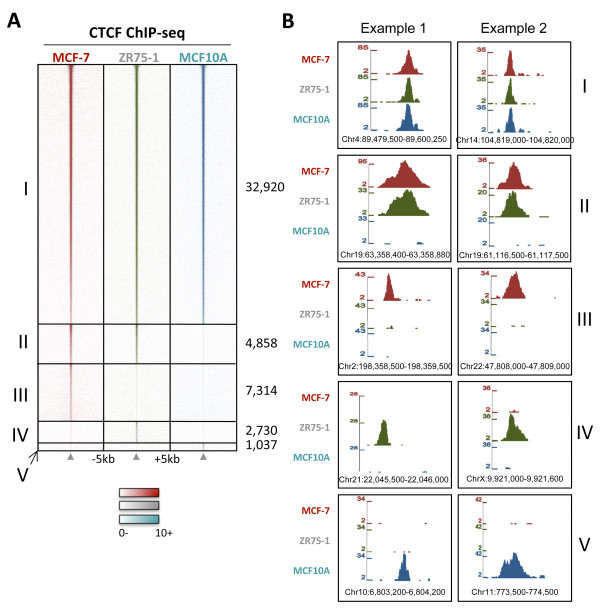
**CTCF can bind uniquely in different breast cell lines**. CTCF binding was mapped in two ER-positive breast cancer cell lines (MCF-7 and ZR75-1) as well as an ER-negative breast cell line (MCF10A). **A**. Heatmap showing CTCF binding profiles in MCF-7, ZR75-1 and MCF10A cells. CTCF binding events that are shared between all three cell lines (I), that are present in only the ER-positive MCF-7 and ZR75-1 cells (II), as well as CTCF binding events are unique to MCF-7 (III) or ZR75-1 (IV) or MCF10A cells (V) are shown. The window represents -/+ 5 kb regions from the centre of the CTCF binding events. **B**. Examples of genomic regions that are: I, common CTCF binding events, II: CTCF binding events present in ER-positive cell lines only, III: MCF-7 unique, IV: ZR75-1 unique, and V: MCF10A-unique CTCF binding events.

As some CTCF binding events overlap with ER and FOXA1 binding events, we assessed whether the CTCF binding profile in an ER-negative cell line, lacking ER and FOXA1 expression, would differ. In order to determine this, CTCF ChIP-seq was performed in the ER-negative breast cell line MCF10A, originally generated from a woman with fibrocystic disease [32]. CTCF was mapped, in duplicate, in MCF10A cells; this resulted in the identification of 39, 995 CTCF-bound genomic loci (Additional File 1). Analysis showed that the majority of CTCF binding events were shared between all three interrogated cell lines and that these common CTCF binding events were generally the strongest bound regions (Figure 4A). However, reproducible differences in CTCF binding among the three breast cell lines were observed (Figure 4A).

A cell-line specific CTCF binding event was defined as a peak identified in both replicates of that cell line and in neither replicate of the other cell lines. This resulted in 7, 314 MCF-7-specific CTCF binding events, 2, 730 ZR75-1-specific CTCF binding events, and 1, 037 MCF10A-specific CTCF binding events (Figure 4A). Examples of these are shown in Figure 4B. In addition, 4, 858 CTCF binding events were identified in both ER-positive cell lines, but not the ER-negative MCF10A cell line (Figure 4A). This overlap is higher than the number of CTCF binding events that were common to only one of the ER-positive cell lines and the ER-negative cell line (795 CTCF binding regions shared between MCF-7 and MCF10A and 1, 354 CTCF binding events shared between ZR75-1 and MCF10A), suggesting a link between CTCF and ER binding.

On the whole, the cell-line specific CTCF binding events were weaker than the common CTCF binding events, but they were reproducible and therefore may contribute to cell-line specific gene expression. Motif analysis was performed on the cell-line unique CTCF binding events to determine if there were any differences in enriched motifs in the cell-line unique CTCF binding events. In the common CTCF binding events, the CTCF motif was the only motif that was enriched. However, the cell-line specific CTCF binding events in all cell lines were enriched for the MYF motif (Additional File 4). The MCF10A-specific CTCF binding regions also showed enrichment for AP-1 and TAL1:TCF3 motifs. The function of these potential binding sites is unknown, but suggests a role for MYF and AP-1 transcription factors in CTCF function.

### Cell-line specific CTCF binding is more likely to overlap with cell-line specific ER binding

**Figure 5:**
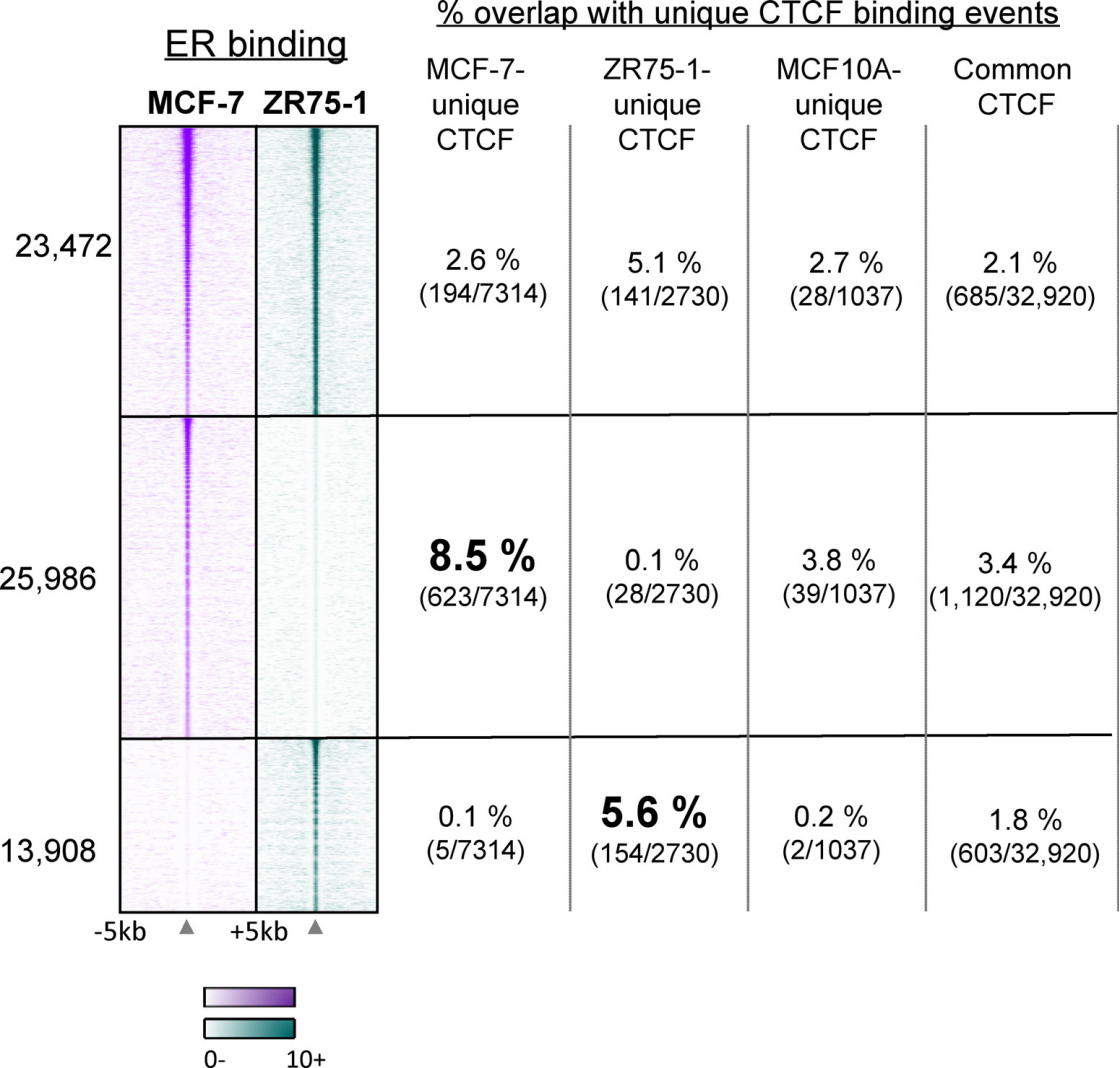
**Cell-line unique CTCF binding events are more likely to overlap with cell-line specific ER binding events**. ER binding was mapped in MCF-7 and ZR75-1 cells using ChIP-seq. Heatmap showing MCF-7- and ZR75-1-unique ER binding events. The window represents -/+ 5 kb regions from the centre of the ER binding events. Shared ER binding events, as well as MCF-7 and ZR75-1 unique ER binding events were overlapped with CTCF binding events that were unique to MCF-7, ZR75-1 or MCF10A cells as well as CTCF binding events that were shared between all three cell lines (Common CTCF). Percentage overlaps are shown.

ER binding profiles differ in different breast cancer cell lines [5]. This may be due to different ER protein levels, different phosphorylation of ER or differences in cofactor levels within the cell lines. In this study we found 23, 472 ER binding events were shared between MCF-7 and ZR75-1 cells (i.e. peaks called in both replicates in both cell lines), 25, 986 ER binding events were specific to MCF-7 cells (peaks called in both MCF-7 replicates and none of the ZR75-1 replicates) and 13, 908 ER binding events could only be identified in ZR75-1 cells (called in both ZR75-1 replicates and none of the MCF-7 replicates) (Figure 5). This shows that only 50 to 60% of ER binding events are shared between two ER-positive cells lines, and generally the overlapping ER binding events are the stronger ER binding events. MCF10A does not express ER and was therefore not included in this analysis.

If CTCF and ER are interacting co-operatively, the cell-line unique CTCF binding events would be more likely to overlap with the cell-line unique ER binding events. We assessed this by overlapping the cell-line unique CTCF binding events with the cell-line unique ER binding events. A larger overlap (8.5%) between MCF-7-specific CTCF and MCF-7-specific ER binding was observed, compared to the overlap between ZR75-1-specific CTCF binding and MCF-7-specific ER binding (0.1%) (Figure 5). Additionally, ZR75-1-specific CTCF binding events were more likely to overlap with ZR75-1-specific ER binding events (5.6%) compared to the other cell-line specific CTCF binding events (0.1% for MCF-7 specific and 0.2% for MCF10A-specific CTCF binding events) (Figure 5). These data suggest that cell-line specific CTCF and ER binding may be functionally related.

### Cell-line unique CTCF binding events are likely to be functional

We asked whether at least some cell line specific CTCF binding events are functional. Although it is difficult to test globally whether ChIP-seq peaks are functional, we hypothesized that if they are, their genomic location should be biased towards genes that are differentially regulated in the corresponding cell line, with respect to other cell lines. To test this hypothesis, we performed gene expression analysis of proliferating MCF-7, ZR75-1 and MCF10A cells, and compared the differentially expressed (DE) genes in each cell line with cell-line specific CTCF binding events.

For each cell line, a list of candidate genes was constructed of genes that were DE in that cell line with respect to both other cell lines (p < 0.01), but not DE between the other two cell lines. This resulted in 2, 503, 2, 140 and 2, 306 genes being included in the analysis for MCF10A, MCF-7 and ZR75-1, respectively. Lists of genes within 20 kb of cell-line unique CTCF peaks were constructed, with 438, 436 and 534 genes found for MCF10A, MCF-7 and ZR75-1, respectively. Fisher's exact test was performed to determine whether DE genes were over-represented in the lists of genes near CTCF peaks. Table 1 shows the results of the tests.

**Table 1:** Cell-line unique CTCF binding events are biased towards genes that are differentially regulated in the corresponding cell line, with respect to other cell lines.

	Genes adjacent to cell-line unique CTCF peaks			
**Genes Differentially Expressed**		**MCF10A**	**MCF-7**	**ZR75-1**
	
	**MCF10A**	p = 4.652e-14	p < 2.2e-16	p = 0.5983
	
	**MCF-7**	p = 0.06637	p < 2.2e-16	p = 0.4189
	
	**ZR75-1**	p = 0.5536	p < 2.2e-16	p = 0.002955

The number of genes differentially expressed in each cell line, with respect to both other cell lines, but not in the other cell lines with respect to each other; the number of genes within 20 kb of a cell-line specific CTCF binding site; and the p-values from Fisher's exact test of the overlaps between those sets. The universe of genes is the 24, 928 distinct genes present on the Illumina gene expression arrays used in the experiment.

Table 1 shows that genes DE in MCF10A are very significantly associated with MCF10A-specific CTCF peaks, but genes DE only in MCF-7 or ZR75-1 are not significantly associated with these peaks. Similarly, genes DE in ZR75-1 cells are significantly associated with CTCF peaks unique to those cells, while genes DE in the other cell lines are not. Unexpectedly, all three cell lines showed a pattern of DE genes being associated with MCF-7 unique peaks. However, the odds ratio for the MCF-7 genes was higher than for the other two cell lines (1.777 for MCF-7 versus 1.558 and 1.565 for MCF10A and ZR75-1), so it is still arguably the case that MCF-7 DE genes are preferentially associated with MCF-7 unique CTCF sites. It may be that the large number of MCF-7 unique CTCF sites simply means that by chance, many genes in each cell line are near at least one site. These results demonstrate that the cell-line unique CTCF binding events are statistically biased towards genes that are differentially expressed in that cell line, suggesting that the CTCF unique sites are functional and are modifying the chromatin to influence gene transcription.

CTCF is a highly conserved protein that has many different roles in a cell. In this study an additional role for CTCF as a transcriptional regulator, in combination with the steroid receptor ER, and the pioneer factor FOXA1, is described. CTCF has previously been shown to be required for hormone-responsive silencing of target genes, together with the nuclear receptors, thyroid hormone receptor and retinoic acid receptor [12,13]. In these studies, mutation of the CTCF binding motif resulted in genes no longer being repressed in response to ligand, indicating that CTCF is required for hormone-responsive silencing of target genes. At these regions it was shown that CTCF was required to recruit corepressors, such as Sin3A and histone deacetylases, in order to silence expression of the target genes [33]. Our study now shows that regions bound by ER and CTCF are enriched near estrogen-regulated genes, and especially estrogen down-regulated genes. It is possible that CTCF is playing a similar role together with ER, and that CTCF is required to recruit co-repressors in order to silence gene transcription in response to estrogen treatment.

Interestingly, the CTCF and thyroid response elements responsible for the synergistic gene silencing between CTCF and thyroid hormone receptor are separated by 160 base pairs [12]. This is similar to what was observed in this study, as the ER, FOXA1 and CTCF binding peaks do not overlap perfectly, but rather, may be shifted to one side. Thus far, CTCF has not been shown to interact directly with the thyroid hormone receptor in *in vitro *pull down assays [12]. This may be due to technical issues or perhaps an additional protein is required in the interaction between CTCF and thyroid hormone receptor. Likewise, it remains to be determined whether ER, FOXA1 and CTCF interact directly or form part of the same complex. As the ER, FOXA1 and CTCF motifs are so clearly enriched in the various categories, it seems likely that these factors bind directly to the DNA and co-operate to regulate target genes.

It has been shown that CTCF binding to target sites flanking the TFF1 locus form a chromatin loop and are required for the TFF1 locus to be estrogen responsive [24]. This study has identified additional estrogen-regulated genes, namely XBP1, GREB1 and NRIP1, that may require CTCF binding to demarcate the estrogen-responsive regions and allow the genes to be estrogen regulated. It is possible that CTCF acts as a barrier insulator at these regions to prevent the spreading of heterochromatin. At other specific regions, CTCF may negatively affect the binding potential of FoxA1 [5]. In addition, a small percentage of genomic regions bound by ER/FOXA1/CTCF (150 out of 2, 301) or ER/CTCF (93 out of 2, 308) are involved in ER chromatin loops [31], supporting the idea that CTCF can form loops together with ER to demarcate estrogen-responsive regions in the genome.

As CTCF binding is not responsive to estrogen or tamoxifen in MCF-7 cells and occurs in the absence of estrogen treatment, CTCF must bind to the chromatin independently of ER. As cell-line specific CTCF binding events are more likely to overlap with cell-line specific ER binding events, CTCF may direct ER binding at these regions thereby acting as a 'licensing factor' for ER. This hypothesis is supported by Zhang et al., who showed that FOXA1 binding, and therefore presumably ER binding, was dependent on CTCF binding to the TFF1 locus [24]. Adding another level of complexity, previous studies have demonstrated that multiple nucleosome position sites within the chromatin are required to direct nucleosome positioning [34,35]. These nucleosome position patterns are necessary for CTCF to bind to insulator regions. It may be thus hypothesised that nucleosome position sites within the genome direct where CTCF binds, which further directs where the pioneer factor FOXA1 binds, ultimately regulating binder binding to the chromatin.

## Conclusions

The evolving role for CTCF in ER biology is complex, but is likely to be multifunctional and possibly influenced by the specific genomic locus. Our data suggests that CTCF not only provides boundaries for accessible and 'protected' transcriptional blocks, but may also influence the actual binding of ER to the chromatin, thereby modulating the estrogen-mediated gene expression changes observed in breast cancer cells.

## Methods

### Cell culture

MCF-7 cells were grown in DMEM containing 10% heat-inactivated FBS, 2 mM L-glutamine, 50 U/ml penicillin and 50 μg/ml streptomycin and ZR75-1 cells were grown in RPMI containing 10% heat-inactivated FBS, 2 mM L-glutamine, 50 U/ml penicillin and 50 μg/ml streptomycin. MCF10A cells were maintained in Mammary Epithelium Cell Growth Medium bullet kit (Clonetics, Lonza, MD, USA), containing mammary epithelium basal medium supplemented with bovine pituitary extract, human epidermal growth factor, hydrocortisone and GA-1000 (Gentamicin Sulfate and Amphotericin-B). All cells were genotyped to ensure their identity using short tandem repeat (STR) PCR. To hormone deprive the cells, cells were grown in steroid-depleted medium for three days and then treated with vehicle (ethanol), 100 nM estrogen or 1μM tamoxifen for 45 minutes and three hours.

### Chromatin immunoprecipitations

The antibodies used were anti-ER (sc-543) from Santa Cruz Biotechnologies and anti-CTCF (07-729) from Millipore. At least four 15 cm dishes of cells were used per chromatin immunoprecipitation (ChIP) and samples were processed according to standard ChIP procedures [36]. The immunoprecipitated DNA was subsequently amplified as previously described for Illumina sequencing [36].

### High-throughput sequencing and enrichment analysis

Sequences generated by the Illumina genome analyzer were aligned against NCBI Build 36.3 of the human genome using MAQ http://maq.sourceforge.net/ with default parameters. Peaks were called using Model-based Analysis for ChIP-Seq (MACS) [27], run using default parameters (except mfold = 30). Data was further analysed using the web-based tool, Galaxy [37].

### Heat map generation

To generate the heat maps of the raw ChIP-sequencing (ChIP-seq) data, CTCF or ER binding peaks were used as targets to centre each window. Each window was divided into 100 bins of 100 bp in size. An enrichment value was assigned to each bin by counting the number of sequencing reads in that bin and subtracting the number of reads in the same bin of an input library. Each data set was normalised to 10 million reads. Data were visualized with Treeview [38].

### Structural correction analysis

To determine whether the overlap between transcription factors (ER, CTCF and FOXA1) was statistically significantly higher than expected by random chance, we applied the genome structural correction statistic of Bickel et al. [29,30]. This conservative statistic takes into account the structure of bound regions across the genome in assessing the significance of overlaps. All comparisons had a p-value of approximately 0 with 10, 000 sampling iterations, so we reject the null hypothesis that the transcription factors' binding sites are unrelated, and conclude that their overlap is statistically significant.

### Motif analysis

Two kilobases of sequence surrounding the summit positions were retrieved for each summit set. For each set, the number of matches to a position weight matrix (PWM) with a similarity score of 85% or more was counted in 100 bp non-overlapping windows across the 2 kb regions on both strands. To determine if the number of PWM matches was significant, 1, 000 randomly permuted versions of the matrix were generated and matches were counted in each window on both strands. The random matrix hits were used to generate a distribution from which an empirical p-value was calculated for each window. Specifically the area under a gaussian density curve for values greater than or equal to the number of PWM matches for the original matrix was calculated. This procedure was repeated for each of the 476 PWM in the JASPAR_CORE_2009 collection.

### Genomic location

Genomic location analysis was performed using CEAS http://ceas.cbi.pku.edu.cn/.

### Gene expression analysis

Total RNA was collected from proliferating cells and RNA was hybridised to Illumina arrays. The Illumina BeadChip (HumanWG-6 v3) bead-level data was pre-processed, log2 transformed and quantile normalised using the beadarray package in Bioconductor. Differential expression analysis was performed using the eBayes measure from the limma R package [39] with a Benjamini & Hochberg multiple test correction procedure [40] to identify statistically significant differentially expressed genes (adjusted p-value < 0.01).

### Statistics

p values were computed using the Chi squared test and the two-tailed students t test using excel, as well as the Fisher's exact test [41].

### Data accession

Data for the ChIP-seq experiments are deposited under ArrayExpress accession number E-MTAB-740.

Reviewer Username: Reviewer_E-MTAB-740, Reviewer Password: tGG21ssp

Data for the gene expression microarrays are deposited under ArrayExpress accession number E-MTAB-739.

Reviewer Username: Reviewer_E-MTAB-739, Reviewer Password: ryLB299a

## Competing interests

The authors declare that they have no competing interests.

## Authors' contributions

CSRI and JSC devised all experiments. CSRI conducted all experiments and data analysis was conducted by CSRI and GDB. The paper was written by CSRI, GDB and JSC. All authors have read and approve of the final manuscript.

## Supplementary Material

Additional file 1 **Illumina sequencing data for all samples included in this study**. CTCF or ER ChIP-sequencing was performed on the different cell lines. Detailed include the number of filtered reads as well as the number of peaks identified using MACS. Click here for file

Additional file 2 **CTCF binding demarcates estrogen-regulated genes**. CTCF and ER binding profiles were mapped by ChIP-sequencing in MCF-7 cells. Examples of three classic estrogen-regulated genes, where ER binding events that regulate expression of the genes are flanked by CTCF binding events, are shown. **A**. The XBP1 genomic locus. **B**. The NRIP1 genomic locus. **C**. The GREB1 genomic locus. Click here for file

Additional file 3 **CTCF binding can co-localise with ER and/or FOXA1 in ZR75-1 cells**. CTCF, ER and FOXA1 binding profiles in ZR75-1 cells were analysed. **A**. Heatmap showing clustered binding signal for ER, FOXA1 and CTCF binding in the ZR75-1 cell line. The heatmap shows regions co-bound by ER/FOXA1/CTCF, or ER/CTCF or FOXA1/CTCF. The window represents -/+ 5 kb regions from the centre of the binding events. **B**. Two examples of genomic loci bound by ER, FOXA1 and CTCF in ZR75-1 cells. **C**. Heatmaps showing enriched motifs (p values are shown) in regions bound by CTCF together with ER and/or FOXA1. Click here for file

Additional file 4 **Motif analysis was performed on the different categories of CTCF binding events**. Heatmaps showing enriched motifs (p values are shown) in the CTCF binding events that are common or unique to the different cell lines. Click here for file
