# Supplementary material for: A co-ordinated interaction between CTCF and ER in breast cancer cells
Source: BMC Genomics. 2011 Dec 5;12:593. doi: 10.1186/1471-2164-12-593 (PMC3248577; doi:10.1186/1471-2164-12-593)
Supplement: Additional file 1 — Illumina sequencing data for all samples included in this study. CTCF or ER ChIP-sequencing was performed on the different cell lines. Detailed include the number of filtered reads as well as the number of peaks identified using MACS. [file 1471-2164-12-593-S1.PDF]

Additional file 1: Illumina sequencing data for all samples included in this study. CTCF or ER ChIP-sequencing was performed on the different cell lines. Detailed below are the number of filtered reads as well as the number of peaks identified using MACS.

CTCF ChIP-sequencing samples	Aligned reads	MACS peaks
MCF-7 vehicle 45 minutes	21,006,949	69,224
MCF-7 estrogen 45 minutes	20,855,204	74,867
MCF-7 4-hydroxytamoxifen 45 minutes	7,016,827	57,209
MCF-7 vehicle 3 hours	27,341,097	60,537
MCF-7 estrogen 3 hours	30,446,660	56,342
MCF-7 4-hydroxytamoxifen 3 hours	29,935,795	69,264
ZR75-1 replicate 1	19,847,141	51,077
ZR75-1 replicate 2	28,477,837	63,132
MCF10A replicate 1	18,067,910	43,106
MCF10A replicate 2	29,473,116	50,606
MCF-7 input	27,858,051	
ZR75-1 input	19,239,636	
MCF10A input	16,122,876	

ER ChIP-sequencing samples	Filtered reads	MACS peaks
MCF-7 replicate 1	26,476,208	77,532
MCF-7 replicate 2	34,107,992	70,880
ZR75-1 replicate 1	27,366,265	62,828
ZR75-1 replicate 2	95,971,691	45,867
