# Supplementary material for: A co-ordinated interaction between CTCF and ER in breast cancer cells
Source: BMC Genomics. 2011 Dec 5;12:593. doi: 10.1186/1471-2164-12-593 (PMC3248577; doi:10.1186/1471-2164-12-593)

Additional file 2: CTCF binding demarcates estrogen-regulated genes. CTCF and ER binding profiles were mapped by ChIP-sequencing in MCF-7 cells. Examples of three classic estrogen-regulated genes, where ER binding events that regulate expression of the genes are flanked by CTCF binding events, are shown. **A**. The XBP1 genomic locus. **B**. The NRIP1 genomic locus. **C.** The GREB1 genomic locus.

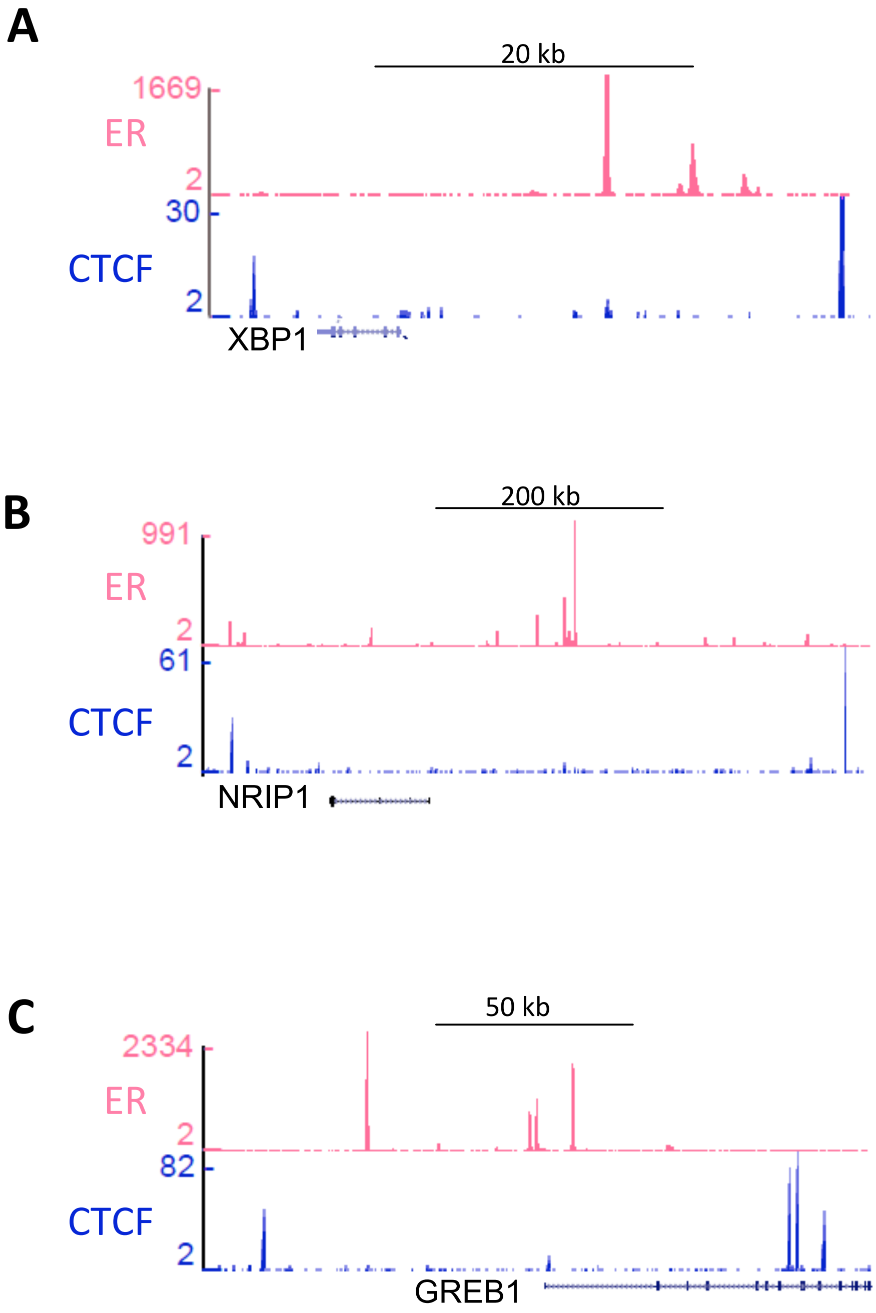

Supplement: Additional file 2 — CTCF binding demarcates estrogen-regulated genes. CTCF and ER binding profiles were mapped by ChIP-sequencing in MCF-7 cells. Examples of three classic estrogen-regulated genes, where ER binding events that regulate expression of the genes are flanked by CTCF binding events, are shown. A. The XBP1 genomic locus. B. The NRIP1 genomic locus. C. The GREB1 genomic locus. [file 1471-2164-12-593-S2.PDF]
