# Supplementary material for: A co-ordinated interaction between CTCF and ER in breast cancer cells
Source: BMC Genomics. 2011 Dec 5;12:593. doi: 10.1186/1471-2164-12-593 (PMC3248577; doi:10.1186/1471-2164-12-593)

Additional file 3. CTCF binding can co-localise with ER and/or FOXA1 in ZR75-1 cells. CTCF, ER and FOXA1 binding profiles in ZR75-1 cells were analysed. **A.** Heatmap showing clustered binding signal for ER, FOXA1 and CTCF binding in the ZR75-1 cell line. The heatmap shows regions co-bound by ER/FOXA1/CTCF, or ER/CTCF or FOXA1/CTCF. The window represents -/+ 5 kb regions from the centre of the binding events. **B**. Two examples of genomic loci bound by ER, FOXA1 and CTCF in ZR75-1 cells. **C**. Heatmaps showing enriched motifs (p values are shown) in regions bound by CTCF together with ER and/or FOXA1

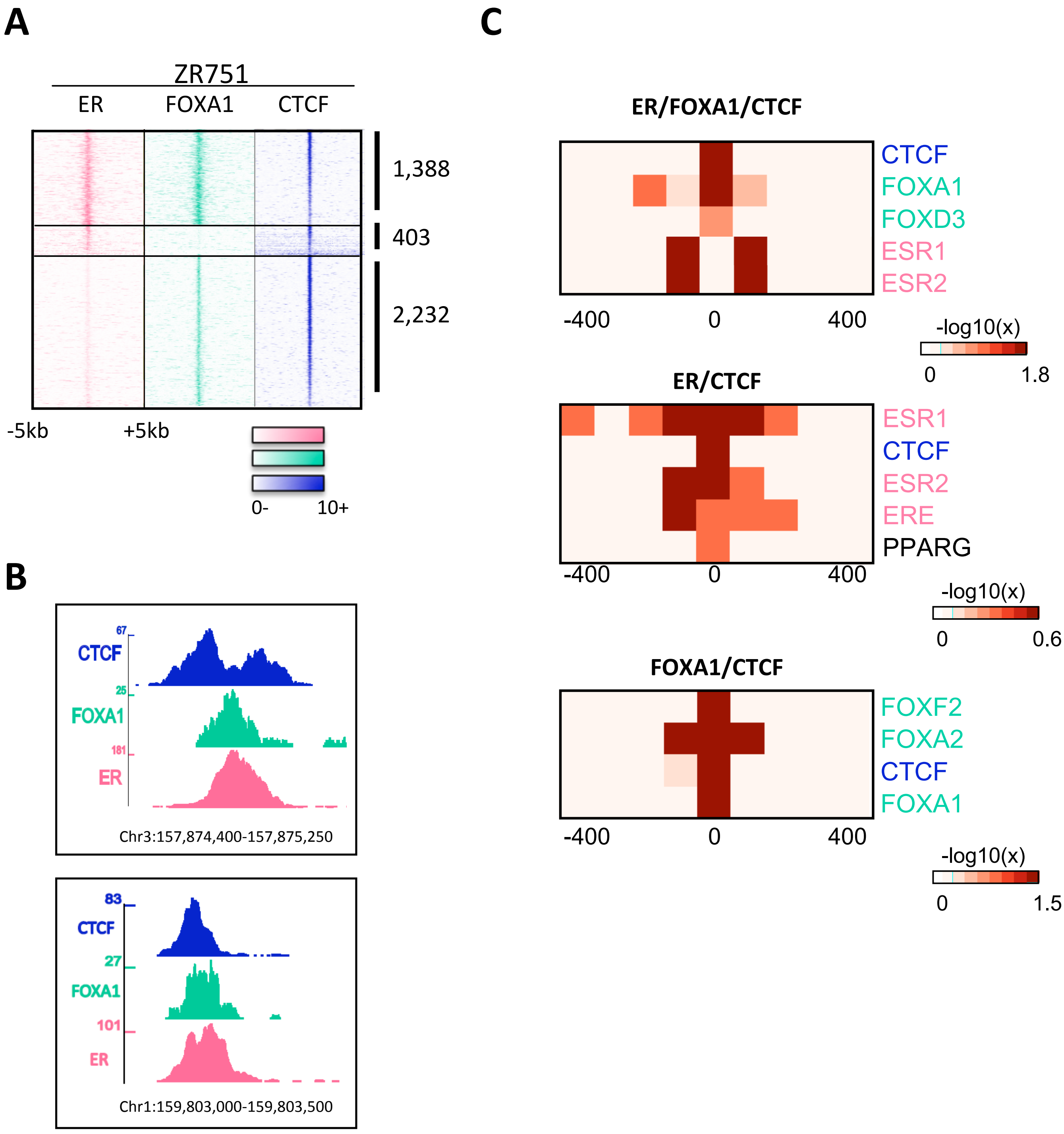

Supplement: Additional file 3 — CTCF binding can co-localise with ER and/or FOXA1 in ZR75-1 cells. CTCF, ER and FOXA1 binding profiles in ZR75-1 cells were analysed. A. Heatmap showing clustered binding signal for ER, FOXA1 and CTCF binding in the ZR75-1 cell line. The heatmap shows regions co-bound by ER/FOXA1/CTCF, or ER/CTCF or FOXA1/CTCF. The window represents -/+ 5 kb regions from the centre of the binding events. B. Two examples of genomic loci bound by ER, FOXA1 and CTCF in ZR75-1 cells. C. Heatmaps showing enriched motifs (p values are shown) in regions bound by CTCF together with ER and/or FOXA1. [file 1471-2164-12-593-S3.PDF]
