# Supplementary material for: A co-ordinated interaction between CTCF and ER in breast cancer cells
Source: BMC Genomics. 2011 Dec 5;12:593. doi: 10.1186/1471-2164-12-593 (PMC3248577; doi:10.1186/1471-2164-12-593)

Additional file 4: Motif analysis was performed on the different categories of CTCF binding events. Heatmaps showing enriched motifs (p values are shown) in the CTCF binding events that are common or unique to the different cell lines.

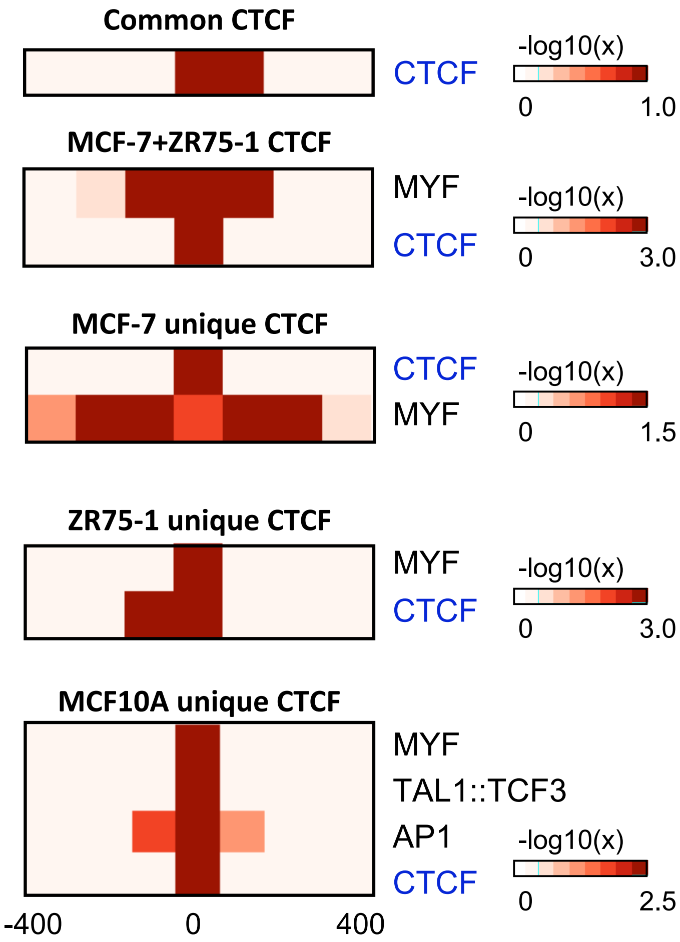

Supplement: Additional file 4 — Motif analysis was performed on the different categories of CTCF binding events. Heatmaps showing enriched motifs (p values are shown) in the CTCF binding events that are common or unique to the different cell lines. [file 1471-2164-12-593-S4.PDF]
